# Viral Transmission and Clinical Features in Asymptomatic Carriers of SARS-CoV-2 in Wuhan, China

**DOI:** 10.3389/fmed.2020.00547

**Published:** 2020-08-18

**Authors:** Fen Tan, Kaige Wang, Jiasheng Liu, Dan Liu, Jianfei Luo, Rui Zhou

**Affiliations:** ^1^Department of Pulmonary and Critical Care Medicine, The Second Xiangya Hospital, Respiratory Disease Research Institute of Hunan Province, Central South University, Changsha, China; ^2^Department of Pulmonary and Critical Care Medicine, West China Hospital, Sichuan University, Chengdu, China; ^3^Department of Gastrointestinal Surgery, Renmin Hospital of Wuhan University, Wuhan, China

**Keywords:** SARS-CoV-2, COVID-19, asymptomatic carrier, transmission ability, clinical features

## Abstract

We report the clinical characteristics, viral shedding duration, and contact tracing for asymptomatic carriers of SARS-CoV-2 in Wuhan, China. The asymptomatic carriers were relatively young (median age: 34.5 years). Chest computed tomography showed no abnormalities. The nasopharyngeal swab was an optimum specimen for RNA testing. The median viral shedding duration was 11.5 days. Notably, 2 months of viral shedding duration were reported in two nurses, which was much longer than previously reported or than usually thought. The transmissibility of SARS-CoV-2 by asymptomatic carriers during the studied period in Wuhan appeared to be weak. Only one patient (1/12) was found to have transmitted the virus to another person. Early asymptomatic carrier detection, isolation, and contact tracing could be useful to mitigate the spread of the disease.

## Introduction

The epidemic of coronavirus disease 2019 (COVID-19) has spread globally and has resulted in more than 600,000 deaths as of late July (1). In the fight against severe acute respiratory syndrome coronavirus 2 (SARS-CoV-2), an increasing number of investigators have begun to be focus on the risk of transmission from asymptomatic carriers. An asymptomatic carrier of SARS-CoV-2 is a person infected with SARS-CoV-2 who does not develop symptoms. According to their current disease state, asymptomatic carriers could be categorized as incubatory carriers in a pre-symptomatic state or convalescent carriers who have already recovered from the disease. Asymptomatic carriers play a critical role in the transmission of infectious diseases, including COVID-19 (2). The reported incidence of asymptomatic infections differs in various regions and time periods. An early report from China stated that only 1% of SARS-CoV-2 infections were asymptomatic (3). The incidence of asymptomatic infections on the “Diamond Princess” ship was 51.7% (4). A high proportion (40.7%) of asymptomatic infections was reported among residents and staff members of nine long-term care facilities in the USA (5). To date, a better understanding of asymptomatic carriers is still urgently needed. We hereby provide a report of healthy asymptomatic carriers based on a review and analysis of their medical records with the ultimate goal of mitigating the spread of COVID-19.

## Methods

We enrolled asymptomatic carriers of SARS-CoV-2 from March 20th, 2020, to April 5th, 2020, in the People's Hospital of Wuhan University. Of the 280 patients with laboratory-confirmed COVID-19, 12 patients who never developed any symptoms throughout the disease course were included in this study. The 12 asymptomatic carriers met the following criteria: (1). Confirmed SARS-CoV-2 infection, with patients having at least two positive results from RNA tests (6). Specimens collected from nasopharyngeal swabs, stool, and urine were analyzed by reverse transcription quantitative PCR to detect SARS-CoV-2 RNA; (2). A lack of related signs or symptoms of COVID-19, including fever and any respiratory symptoms during the entire hospitalization and the 14-day post-discharge isolation period. When SARS-CoV-2 infection was confirmed, the patients were hospitalized in the isolation ward until they met the discharge criteria. Discharge criteria was two consecutive negative results on RNA tests separated by at least a 24-h interval for nasopharyngeal swabs, stool, and urine. During hospitalization, all patients were treated with Arbidol (1,200 mg, three times per day, oral), and RNA testing was repeated every 3 days. After discharge, the patients were quarantined for 14 days in an isolated observation area and underwent viral RNA testing every week. When the test for viral RNA was confirmed to be positive in these patients, any person with whom the patients had contact was strictly quarantined for 14 days and tested for the presence of viral RNA. We analyzed all the clinical features, including the laboratory and radiographic findings. Data are presented as the medians ± interquartile ranges (IQRs) for continuous variables. The study was approved by the People's Hospital of Wuhan University Ethics Committee (No. WDRY2020-K068). Written informed consent was obtained from all the patients for the publication of any potentially identifiable data included in this article.

## Results

The proportion of patients from March 20th, 2020, to April 5th, 2020, in the People's Hospital of Wuhan University who were asymptomatic was 4.3%. The demographic and clinical characteristics of the 12 asymptomatic patients are shown in Table 1. All the patients were Wuhan residents. Seven patients were screened for viral RNA because of a definite history of exposure to confirmed patients, and two of the seven were nurses who had cared for COVID-19 patients in a front-line hospital. Four patients were screened for RNA when they were hospitalized due to an active disease, including acute pancreatitis, ectopic pregnancy, coronary heart disease, and hepatocellular carcinoma. One patient was screened for viral infection during a pre-employment physical examination and did not have a definite history of exposure. The patients had a median age of 34.5 years (IQR 29.0–43.0); nine of them were males and three of them were females.

**Table 1:** Summary of clinical features and laboratory findings.

	**References**	**Patient 1**	**Patient 2**	**Patient 3**	**Patient 4**	**Patient 5**	**Patient 6**	**Patient 7**	**Patient 8**	**Patient 9**	**Patient 10**	**Patient 11**	**Patient 12**
Age	NA	35	43	45	30	27	22	32	34	29	42	52	64
Sex	NA	Male	Male	Male	Male	Male	Male	Female	Female	Female	Male	Male	Male
Occupation	NA	Office worker	Office worker	Office worker	Doctor	Office worker	Student	Office worker	Nurse	Nurse	Office worker	Office worker	Office worker
Contact history	NA	No	No	Yes	Yes	Yes	Yes	No	Yes	Yes	No	Yes	No
Transmission to contact	NA	No	No	No	No	No	Mother	No	No	No	No	No	No
Nasopharyngeal swabs	NA	+	+	+	+	+	+	+	+	+	+	+	+
Stool	NA	+	–	–	–	–	+	+	+	+	+	–	–
Urine	NA	–	–	–	–	–	+	–	–	–	–	–	–
Viral shedding duration	NA	11	8	25	11	11	12	7	70	65	9	14	14
Interval from contact to RNA testing	NA	NA	NA	46	20	24	14	NA	3	6	NA	57	NA
Comorbidity	NA	No	DM	No	No	No	No	No	No	No	CHD	No	HCC
Active diseases	NA	Acute pancreatitis	No	No	No	No	No	Ectopic pregnancy	No	No	CHD	No	HCC
WBC × 10^9^/L	3.5–9.5	6.1	7.8	6.34	7.8	7.29	7.0	12.97	4.37	4.49	6.92	8.09	2.18
Neutrophils × 10^9^/L	1.8–6.3	4.2	4.8	4.04	3.88	4.42	3.78	10.05	2.1	2.12	3.95	2.88	1.39
Lymphocytes × 10^9^/L	1.1–3.2	1.01	2.32	1.71	2.79	2.05	2.46	1.97	1.59	1.86	2.22	4.09	0.43
CRP, mg/L	<5	>200	<5	<5	<5	<5	<5	81	<5	<5	<5	<5	13
IgG^*^, AU/mL	<10	175.4	55.75	45.34	393.3	62.6	149.6	104	129	21.55	87.65	189.2	1,041
IgM^*^, AU/mL	<10	1.52	1.08	15.01	24.58	2.8	3.77	3.59	4.61	6.74	9.03	8.6	4.7
LDH, U/L	120–250	491	154	137	135	204	136	190	121	146	287	160	233
D-dimer, mg/L	0–0.55	9.54	0.21	0.14	0.21	0.1	0.27	0.8	0.15	NA	9.32	NA	NA

*DM, diabetes mellitus; CHD, coronary heart disease; HCC, hepatocellular carcinoma; WBC, white blood cell; CRP, C-reactive protein; LDH, lactate dehydrogenase; NA, not available*.

* *IgG refers to antibody against SARS-CoV-2*.

* *IgM refers to antibody against SARS-CoV-2*.

The retrospective results of RNA testing on specimens, including nasopharyngeal swabs, stool samples, and urine samples, during the entire illness duration are shown in Table 1. Nasopharyngeal swabs had the highest positive rates (12 of 12; 100%), followed by stool samples (6 of 12; 50%) and urine samples (1 of 12; 8.6%). Viral serological test results for IgM and IgG antibodies against SARS-CoV-2 are shown in Table 1. The IgG levels of all the patients were elevated (100%), and the IgM levels of two patients were slightly elevated (16.7%). Eight patients without active disease presented with normal laboratory markers, including white blood cell count and levels of C-reactive protein, lactate dehydrogenase, and D-dimer. Chest computed tomography was normal in all patients as well. Four patients who were hospitalized for active primary diseases had relevant changes in laboratory tests (Table 1). For example, both patient 1 and patient 7 presented with increased C-reactive protein levels because of acute pancreatitis and ectopic pregnancy surgery, respectively.

**Figure 1:**
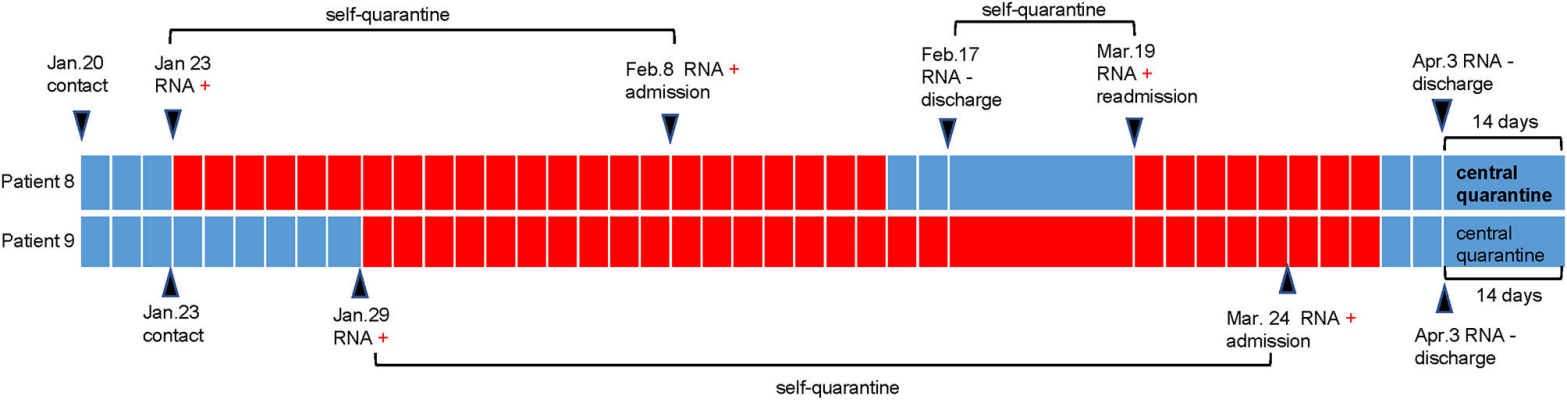
Disease course in patient 8 and patient 9. As shown in the schematic, patient 8 and patient 9 (two nurses) performed their first RNA testing within 1 week after contact with the infected patient. Then they were self-quarantined until admission to hospital. During the period of self-quarantine and central quarantine, they were tested with nasopharyngeal swabs once a week. During the period of hospitalization, they were tested with nasopharyngeal swabs every 3 days. Patient 8 presented with recurrent positivity of RNA testing and was re-admitted to hospital.

The duration from the first confirmed positive RNA test result to a confirmed negative RNA test result is defined as the viral shedding duration (7). The median shedding duration was 11.5 days (IQR 9.0–14.0), ranging from 7 to 70 days. Notably, the shedding duration in the two nurses (patient 8 and patient 9) was more than 2 months (Table 1), which was longer than previously reported or usually thought. We retrospectively tracked the disease course in these two nurses, and the details are shown in Figure 1. We found that these two nurses underwent viral RNA testing within 1 week after exposure to confirmed patients. The intervals from confirmed contact to the first RNA test in these two patients were 3 days and 6 days, which were much shorter than those in the other patients (Table 1). Additionally, patient 8 presented with recurrent positivity for viral RNA during the self-quarantine period after the first discharge. After performing contact tracing for all patients, only one patient was found to have transmitted the virus; he passed the virus to his mother, who developed mild COVID-19. Persons with whom the other 11 patients came into contact were not infected; either no symptoms were observed or screening viral RNA tests were negative during the 14-day isolation period.

## Discussion

SARS-CoV-2 is recognized as being much more transmissible than both SARS-CoV and Middle East respiratory syndrome coronavirus (8). Asymptomatic carriers of SARS-CoV-2 have received increasing attention (7, 9). In this report, we describe some asymptomatic carriers of the disease. Healthy asymptomatic carriers are more likely to be younger (median age: 34.5 years) than symptomatic hospitalized patients (median age: 56.0 years) (10), which is consistent with some recent studies (11). RNA tests of nasopharyngeal swabs were 100% positive, which is much higher than the positive rate in stool and urine specimens in asymptomatic carriers, suggesting that nasopharyngeal swabs could be the optimum samples. However, the nasopharyngeal swabs test was reported to deliver false negatives because of sample collection and the operating procedures in some studies (11). All asymptomatic carriers presented with elevated IgG antibody levels, while the IgM antibody levels were slightly elevated in a few patients in our study. The SARS-CoV-2 specific IgG and IgM yielded different responses depending on disease course. IgG usually maintains at high levels during a long period (12), while IgM usually wanes rapidly (13). IgG also reported to be seronegative in some asymptomatic individuals (7). Undoubtedly, the immune responses play a key role in the onset of COVID-2019. Hence, more immunological studies of asymptomatic carriers are needed urgently.

Prolonged viral shedding has been reported to be associated with fatal outcomes of severe influenza A (H7N9) infection (14). In COVID-19 non-survivors, the virus could be detected up until death. The viral shedding duration is a critical indicator of prognosis in symptomatic patients (10). It is estimated that viral shedding from asymptomatic carriers contributed to early transmission (15). In a previous retrospective cohort study, the median range of the duration of viral shedding among hospitalized patients was 12–20 days (10). The longest observed duration of viral shedding in survivors was 49 days (16). In study of 37 asymptomatic individuals (Chongqing, China), shedding duration was reported as 19 days (7). In our study, the median shedding duration of asymptomatic carriers was 11.5 days. Notably, the longest duration of viral shedding in two nurses was longer than 2 months. This is the longest viral shedding duration reported to date. When tracing the entire disease course, we found that the first RNA testing was performed much earlier in these two nurses than in other patients due to their confirmed exposure history. Besides early detection of RNA, the prolonged virus shedding duration in asymptomatic carriers is predicted to be associated with the frequency and quality of specimen collection (7). In addition, evidence of virus shedding duration only evaluated by reverse transcription quantitative PCR is limited, for RNA testing cannot distinguish whether the virus is alive or dead (17). The virus viability assessment in patients should be considered in future studies. One of the nurses developed a recurrent RNA positivity after discharge. Nasopharyngeal swabs, while effective, are unable to account for the possibility of reinfection and can also deliver false negative results (18). Our findings suggested that asymptomatic carriers could remain free from symptoms while carrying the virus for an extended period. This may provide evidence of the strong potential for transmission by asymptomatic carriers. Several studies have indicated that transmission of SARS-CoV-2 by asymptomatic carriers is implicated in crowds and family outbreaks in Wuhan, from December, 2019 to January, 2020 (19, 20). However, according to the results of contact tracing collected from March 20th, 2020, to April 5th, 2020 in our study, it was found that the transmissibility of the virus in asymptomatic carriers was weak, which is consistent with previous studies that suggested that the transmission risk is not high when patients are asymptomatic (21, 22). Additionally, it was reported that all 455 contacts who were exposed to the asymptomatic carriers in Guangdong, China, did not develop SARS-CoV-2 infection (23). Thus, the transmissibility of the virus in asymptomatic carriers might be weak. It is worth noting that the low transmissibility is probably related to the strict control measures implemented since February in Wuhan. More evidence is needed to clarify the transmissibility of the virus in asymptomatic carriers in the future. The collection of data from a larger cohort would enable researchers to more comprehensively investigate this issue.

In conclusion, viral RNA can be detected in asymptomatic carriers over a long period. The transmissibility of the virus in asymptomatic carriers from Wuhan, where strict control measures were implemented, was not as high as expected. Early asymptomatic carrier detection, isolation, and contact tracing would be useful to mitigate the spread of COVID-19. This report will hopefully provide a better understanding of the transmissibility of the virus in asymptomatic carriers of COVID-19.

## Data Availability Statement

All datasets generated for this study are included in the article/supplementary material.

## Ethics Statement

Written informed consent was obtained from the [individual(s) and/or minor(s)' legal guardian/next of kin] for the publication of any potentially identifiable images or data included in this article.

## Author Contributions

RZ, DL, and JLu directed the whole study to go on. KW, JLu, and JLi gathered information of all patients. FT and KW analyzed the data. FT drawed the table and the figure. FT wrote the manuscript. RZ, DL, and JLu reviewed and amended the manuscript. All authors contributed to the article and approved the submitted version.

## Conflict of Interest

The authors declare that the research was conducted in the absence of any commercial or financial relationships that could be construed as a potential conflict of interest.

## Abbreviations

COVID-19: coronavirus disease 2019
SARS-CoV-2: severe acute respiratory syndrome coronavirus 2
IQR: interquartile range.
